# Smoking Status and Survival in Patients With Early-Stage Primary Cutaneous Melanoma

**DOI:** 10.1001/jamanetworkopen.2023.54751

**Published:** 2024-02-06

**Authors:** Katherine M. Jackson, Peter C. Jones, Laura M. Fluke, Trevan D. Fischer, John F. Thompson, Alistair J. Cochran, Stacey L. Stern, Mark B. Faries, Dave S. B. Hoon, Leland J. Foshag

**Affiliations:** 1 Department of Surgical Oncology, Saint John’s Cancer Institute at Providence Saint John’s Health Center, Santa Monica, California; 2 Melanoma Institute Australia, Sydney, Australia; 3 Department of Pathology and Laboratory Medicine, University of California, Los Angeles; 4 Translational Molecular Medicine and Biostatistics, Saint John’s Cancer Institute at Providence Saint John’s Health Center, Santa Monica, California; 5 The Angeles Clinic and Research Institute, Los Angeles, California

## Abstract

**Importance:**

While smoking is associated with a decreased incidence of cutaneous melanoma, the association of smoking with melanoma progression and death is not well defined.

**Objective:**

To determine the association of smoking with survival in patients with early-stage primary cutaneous melanoma.

**Design, Setting, and Participants:**

This cohort study performed a post hoc analysis of data derived from the randomized, multinational first and second Multicenter Selective Lymphadenectomy Trials (MSLT-I and MSLT-II). Participants were accrued for MSLT-I from January 20, 1994, to March 29, 2002; MSLT-II, from December 21, 2004, to March 31, 2014. Median follow-up was 110.0 (IQR, 53.4-120.0) months for MSLT-I and 67.6 (IQR, 25.8-110.2) months for MSLT-II. Patients aged 18 to 75 years with clinical stages I or II melanoma with a Breslow thickness of 1.00 mm or greater or Clark level IV to V and available standard prognostic and smoking data were included. Analyses were performed from October 4, 2022, to March 31, 2023.

**Exposure:**

Current, former, and never smoking.

**Main Outcomes and Measures:**

Melanoma-specific survival of patients with current, former, and never smoking status was assessed for the entire cohort and for nodal observation and among subgroups with sentinel lymph node biopsy (SLNB)–negative and SLNB-positive findings.

**Results:**

Of 6279 included patients, 3635 (57.9%) were men, and mean (SD) age was 52.7 (13.4) years. The most common tumor location was an extremity (2743 [43.7%]), and mean (SD) Breslow thickness was 2.44 (2.06) mm. Smoking status included 1077 (17.2%) current, 1694 (27.0%) former, and 3508 (55.9%) never. Median follow-up was 78.4 (IQR, 30.5-119.6) months. Current smoking was associated with male sex, younger age, trunk site, thicker tumors, tumor ulceration, and SLNB positivity. Current smoking was associated with a greater risk of melanoma-associated death by multivariable analysis for the entire study (hazard ratio [HR], 1.48 [95% CI, 1.26-1.75]; *P* < .001). Former smoking was not. The increased risk of melanoma-specific mortality associated with current smoking was greatest for patients with SLNB-negative melanoma (HR, 1.85 [95% CI, 1.35-2.52]; *P* < .001), but also present for patients with SLNB-positive melanoma (HR, 1.29 [95% CI, 1.04-1.59]; *P* = .02) and nodal observation (HR, 1.68 [95% CI, 1.09-2.61]; *P* = .02). Smoking at least 20 cigarettes/d doubled the risk of death due to melanoma for patients with SLNB-negative disease (HR, 2.06 [95% CI, 1.36-3.13]; *P* < .001).

**Conclusions and Relevance:**

The findings of this cohort study suggest that patients with clinical stage I and II melanoma who smoked had a significantly increased risk of death due to melanoma. Smoking status should be assessed at time of melanoma diagnosis and may be considered a risk factor for disease progression.

## Key Points

**Question:**

Is there an association between smoking and outcomes of patients with clinically node-negative cutaneous melanoma?

**Findings:**

In this cohort study of 6279 patients, current smoking was associated with shorter melanoma-specific survival compared with never smoking, but former smoking was not. Smoking 20 or more cigarettes per day was associated with a doubled risk of death in patients with pathologic node-negative melanoma.

**Meaning:**

These findings suggest that smoking at the time of melanoma diagnosis is associated with increased lymph node metastases and a greater risk of melanoma-specific mortality.

## Teaser

This cohort study assesses the association of smoking with survival among patients with clinical stage I and II melanoma who participated in the first and second Multicenter Selective Lymphadenectomy Trials.

## Introduction

The role of smoking in carcinogenesis is well accepted.^1^ Accordingly, smoking imparts an increased risk of developing multiple types of malignant neoplasms, including those of the lung, bladder, and head and/or neck.^2^ However, studies have failed to show an increased risk of melanoma development in individuals who smoke.^3^ Paradoxically, multiple reports have demonstrated a decreased incidence of melanoma in patients who smoke.^4,5,6^ Furthermore, the findings of some studies suggest an inverse dose-dependent effect, with an increased number of years and/or cigarettes smoked imparting additional protection against development of melanoma.^7,8,9^

In patients with established melanoma, data regarding smoking as an independent prognostic factor for melanoma-specific survival (MSS) are limited and somewhat conflicting. Multiple studies^4,10,11^ have suggested that smoking does not significantly affect sentinel lymph node biopsy (SLNB) positivity or MSS, while others^12,13,14,15^ demonstrate an association of smoking with an increased risk of melanoma metastasis and decreased survival. One study^10^ identified smoking as an independent prognostic factor, but this did not reach statistical significance (*P* = .07), and it was concluded that “smoking need not be considered as an independent stratification criterion.” Recently, Gibson et al^4^ evaluated over 7000 patients and demonstrated a decreased overall survival but not a decreased MSS in smokers compared with nonsmokers.

Previous studies^12,16,17^ have demonstrated that smoking is associated with thicker and ulcerated primary melanoma tumors and an increased incidence of SLNB positivity, which are all strong negative prognostic factors. The few studies that have shown smoking to be independently associated with diminished survival^12,18,19^ did not include ulceration in the multivariate analysis. Thus, it is unclear whether the association of smoking with tumor ulceration and stage contributed to the correlation with decreased survival. Altogether, the effect of smoking on melanoma outcomes is inconsistent across studies, and melanoma may be an atypical cancer for which smoking’s effects on tumor initiation and subsequent propagation diverge.

We undertook an analysis of patients entered in the first and second Multicenter Selective Lymphadenectomy Trials (MSLT-I and MSLT-II) to address the association of smoking with survival in patients with clinical stage I and II melanoma. Using the screening phase of MSLT-II and the complete MSLT-I cohort, Jones et al^16^ previously reported that SLNB positivity was increased in patients who smoke. In the present study, we report smoking-associated survival data in the completed MSLT-I and MSLT-II datasets. To our knowledge, the present study is one of the largest examining the association of smoking with outcomes among patients with clinical stage I and II cutaneous melanoma.

## Methods

### Study Design, Setting, and Patient Population

This study followed the Strengthening the Reporting of Observational Studies in Epidemiology (STROBE) reporting guideline for cohort studies. Participants provided written informed consent for participation in the clinical trials and any subsequent analyses, and all data were deidentified. The trials were approved by each participating institution’s Institutional Review Board.

A post hoc analysis was performed on data derived from patients enrolled in MSLT-I^20^ and MSLT-II.^21^ Both MSLT-I and MSLT-II are prospective international trials evaluating methods of regional draining lymph node management in patients with clinically localized primary cutaneous melanomas. Participants in MSLT-I were accrued from January 20, 1994, through March 29, 2002, and in MSLT-II from December 21, 2004, through March 31, 2014. Treatment by wide local excision (WLE) alone was compared with WLE plus SLNB in MSLT-I, followed by completion lymph node dissection (CLND) for patients found to have SLNB metastases. The trial included patients with primary tumors 1 mm or thicker or thinner tumors with invasion to at least Clark level IV.^22^ In MSLT-II, patients were enrolled prior to SLNB during a screening phase, and patients with nodal metastases identified on SLNB findings were assigned to undergo either CLND or observation plus nodal ultrasonography during a randomization phase.^23^ For inclusion in MSLT-II, primary tumors were at least 1.2 mm in thickness and Clark level III, with invasion to at least Clark level IV, or ulcerated. Patients who were found to have SLN metastases by means of standard pathologic evaluation or by multimarker quantitative reverse transcriptase–polymerase chain reaction analysis (RT-PCR) were eligible for randomization.

After primary and regional lymph node surgery, adjuvant systemic therapy prior to recurrence was uncommon during that period of the study. Per both trial protocols, patients were monitored closely every 3 to 4 months with a history and physical examination for the first few years and then annually for up to 10 years after melanoma diagnosis.^22,24^ The median follow-up for MSLT-I was 110.0 (IQR, 53.4-120.0) months; for MSLT-II was 67.6 (IQR, 25.8-110.2) months.

### Patient Groups

**Figure 1.**
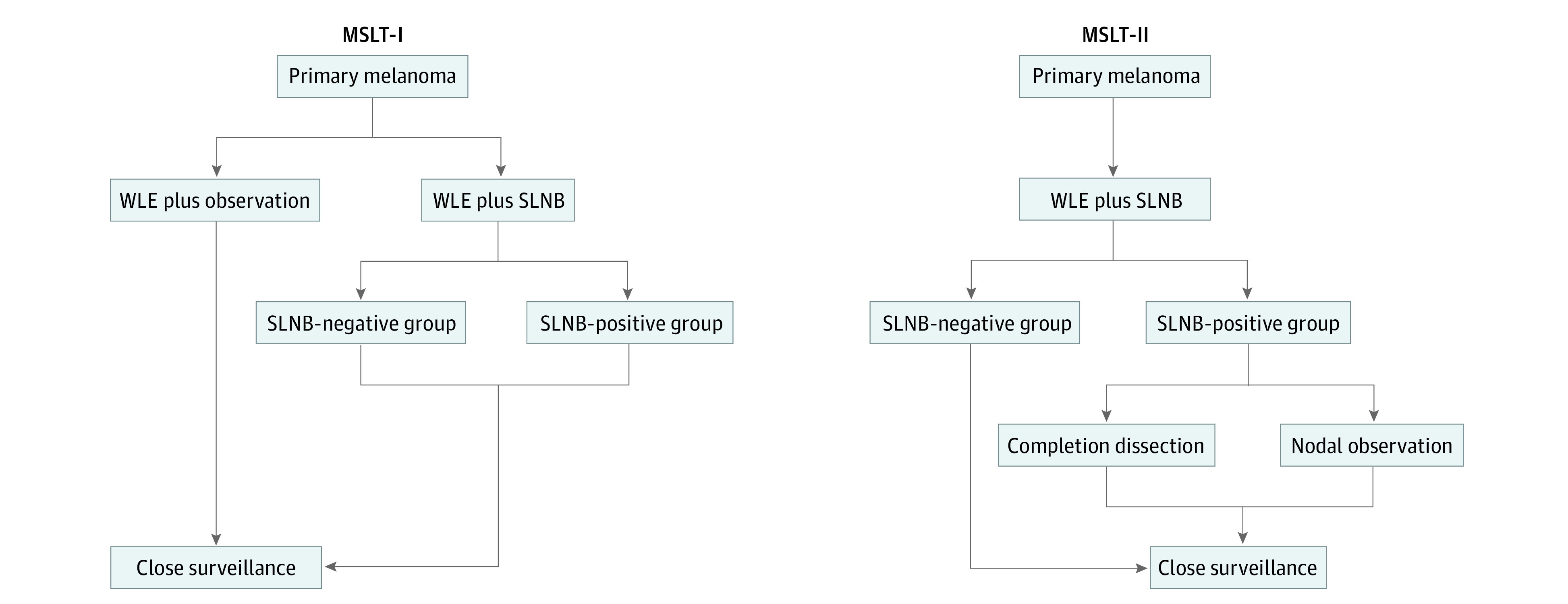
Patient Randomization Schemes From the First and Second Multicenter Selective Lymphadenectomy Trials (MSLT-I and MSLT-II) SLNB indicates sentinel lymph node biopsy; WLE, wide local excision.

Because both MSLT trials had varied initial treatment pathways, as some patients in MSLT-I had no SLNB performed, patients were grouped according to completion of SLNB and subsequent nodal status (Figure 1). The association of smoking status and survival was assessed for 3 groups. Group 1 (SLN plus observation) included patients who did not undergo SLNB in MSLT-I. Group 2 included patients from both MSLT-I and MSLT-II who underwent SLNB with negative findings (hereinafter referred to as the SLNB-negative group). Group 3 included patients from MSLT-I and MSLT-II who underwent SLNB with tumor-positive nodes, by either histologic evaluation or RT-PCR (hereinafter referred to as the SLNB-positive group). In MSLT-I, all patients in the SLNB-positive group underwent CLND, whereas in MSLT-II, half of patients in the SLNB-positive group underwent CLND, and half underwent observation.

### Smoking Status

Smoking status was recorded at a single point at trial enrollment. Patients were categorized as current, former, or never smokers based on self-reporting via either a paper questionnaire or an in-person intake interview. Former smokers had quit smoking at any time prior to trial entry. Smoking included tobacco smoke inhaled via a cigarette, a pipe, a cigar, or another source. For cigarette smokers, cigarettes per day and years smoked were recorded.

### Primary Tumor Parameters and Patient Inclusion

Primary tumor parameters were assessed and confirmed via pathologic review by the trial pathologists. Included patients had known values for all standard demographic and clinical prognostic factors, including age, sex, Breslow thickness, ulceration, primary site, and surgical treatment, as well as known smoking status. Patients with incomplete data were excluded from all reported analyses.

### Statistical Analysis

Data were analyzed from October 4, 2022, to March 31, 2023. Demographic and clinicopathologic factors examined in this study included age, sex, ulceration, Breslow thickness, primary site, SLNB status, and smoking status. These factors were compared among the 3 smoking groups (current, former, and never) using the χ^2^ test for the categorical variables and the Wilcoxon rank sum test for the continuous variables such as age and Breslow thickness. Multivariable analysis was conducted for all factors associated with MSS. We used SAS software, version 9.3 (SAS Institute Inc), for all analyses. A 2-sided *P* ≤ .05 was considered significant.

## Results

### Patient Demographic Characteristics and Clinicopathologic Features by Smoking Status

Of the 6964 patients enrolled in MSLT-I (n = 2001) and MSLT-II (n = 4963), 6279 patients (90.2%) had smoking status and all variables available for analysis and were included in this study. The median follow-up period was 78.4 (IQR, 30.5-119.6) months. Demographic characteristics and clinicopathologic features of the entire cohort stratified by current, former, and never smoking status are shown in Table 1. In terms of race and ethnicity, 6149 patients (97.9%) were non-Hispanic White. The mean (SD) patient age was 52.7 (13.4) years; 2644 (42.1%) were women and 3635 (57.9%) were men. The most common tumor locations were the extremity (2743 [43.7%]) and trunk (2587 [41.2%]). Mean (SD) Breslow thickness was 2.44 (2.06) mm, and overall ulceration was present in 1946 patients (31.0%). There were 1077 (17.2%) current, 1694 (27.0%) former, and 3508 (55.9%) never smokers. A higher proportion of current and former smokers were men (current: 648 [60.2%]; former: 1139 [67.2%]; never: 1848 [52.7%]; *P* < .001). Current smokers were, on average, younger (mean [SD] age, 48.0 [12.4] years), whereas former smokers were, on average, older (mean [SD] age, 56.6 [12.0] years) than nonsmokers (mean [SD] age, 52.2 [13.8] years; *P* < .001). Smokers more frequently presented with melanoma of the trunk (478 [44.4%]) and had the lowest frequency of head and/or neck melanoma (current: 141 [13.1%]; former: 279 [16.5%]; never: 529 [15.1%]; *P* = .002). Current smokers had primary tumors with a greater mean (SD) Breslow thickness (current: 2.61 [2.13] mm; former: 2.50 [1.82] mm; never: 2.36 [2.15] mm; *P* = .001) and a higher incidence of ulceration (current: 413 [38.4%]; former: 529 [31.2%]; never: 1004 [28.6%]; *P* < .001). Current smokers had a higher proportion of positive SLNB findings (current: 441 [40.9%]; former: 588 [34.7%]; never: 1331 [37.9%]; *P* < .001). There was a higher proportion of current and former smokers in MSLT-I (361 [19.8%] and 624 [34.3%], respectively) compared with MSLT-II (716 [16.1%] and 1070 [24.0%], respectively) (*P* < .001). When evaluated by decade, the proportion of current and former smokers decreased from 266 (19.4%) and 488 (35.5%), respectively, in the 1990s to 221 (15.9%) and 337 (24.3%), respectively, in 2010 to 2014 (*P* < .001).

**Table 1.** Demographic Characteristics by Smoking Status

Characteristic	Smoking status^a^				*P* value
	Current (n = 1077 [17.2])	Former (n = 1694 [27.0])	Never (n = 3508 [55.9])	All (N = 6279 [100])	
Sex					
Women	429 (39.8)	555 (32.8)	1660 (47.3)	2644 (42.1)	<.001
Men	648 (60.2)	1139 (67.2)	1848 (52.7)	3635 (57.9)	
Age at trial entry, y					
Mean (SD)	48.0 (12.4)	56.6 (12.0)	52.2 (13.8)	52.7 (13.4)	<.001
Median (range)	48.6 (18-76)	58.2 (18-77)	53.4 (18-81)	54 (18-81)	
Primary site					
Extremity	458 (42.5)	691 (40.8)	1594 (45.4)	2743 (43.7)	.002
Head and/or neck	141 (13.1)	279 (16.5)	529 (15.1)	949 (15.1)	
Trunk	478 (44.4)	724 (42.7)	1385 (39.5)	2587 (41.2)	
Breslow thickness, mm					
Mean (SD)	2.61 (2.13)	2.50 (1.82)	2.36 (2.15)	2.44 (2.06)	.001
Median (range)	2.00 (0.12-28.0)	2.00 (0.28-19.00)	1.75 (0.08-42.00)	1.85 (0.08-42.00)	
Breslow thickness, mm					
<1.00	63 (5.8)	120 (7.1)	416 (11.9)	599 (9.5)	<.001
1.00-1.99	452 (42.0)	709 (41.9)	1542 (44.0)	2703 (43.0)	
2.00-3.99	388 (36.0)	610 (36.0)	1096 (31.2)	2094 (33.3)	
≥4.00	174 (16.2)	255 (15.1)	454 (12.9)	883 (14.1)	
Ulceration					
Present	413 (38.4)	529 (31.2)	1004 (28.6)	1946 (31.0)	<.001
Absent	664 (61.7)	1165 (68.8)	2504 (71.4)	4333 (69.0)	
SLNB status					
No SLNB	135 (12.5)	260 (15.3)	346 (9.9)	741 (11.8)	<.001
SLNB negative	501 (46.5)	846 (49.9)	1831 (52.2)	3178 (50.6)	
SLNB positive	441 (40.9)	588 (34.7)	1331 (37.9)	2360 (37.6)	
MSLT^b^					
MSLT-I	361 (19.8)	624 (34.3)	835 (45.9)	1820 (29.0)	<.001
MSLT-II	716 (16.1)	1070 (24.0)	2673 (59.9)	4459 (71.0)	

Abbreviations: MSLT, Multicenter Selective Lymphadenectomy Trial; SLNB, sentinel lymph node biopsy.

^a^ Unless otherwise indicated, data are expressed as No. (%) of patients given in column heading. Percentages have been rounded and may not total 100.

^b^ Percentages for smoking status groups are calculated based on row totals.

### Association of MSS With Smoking Status by Nodal Groups

**Figure 2.**
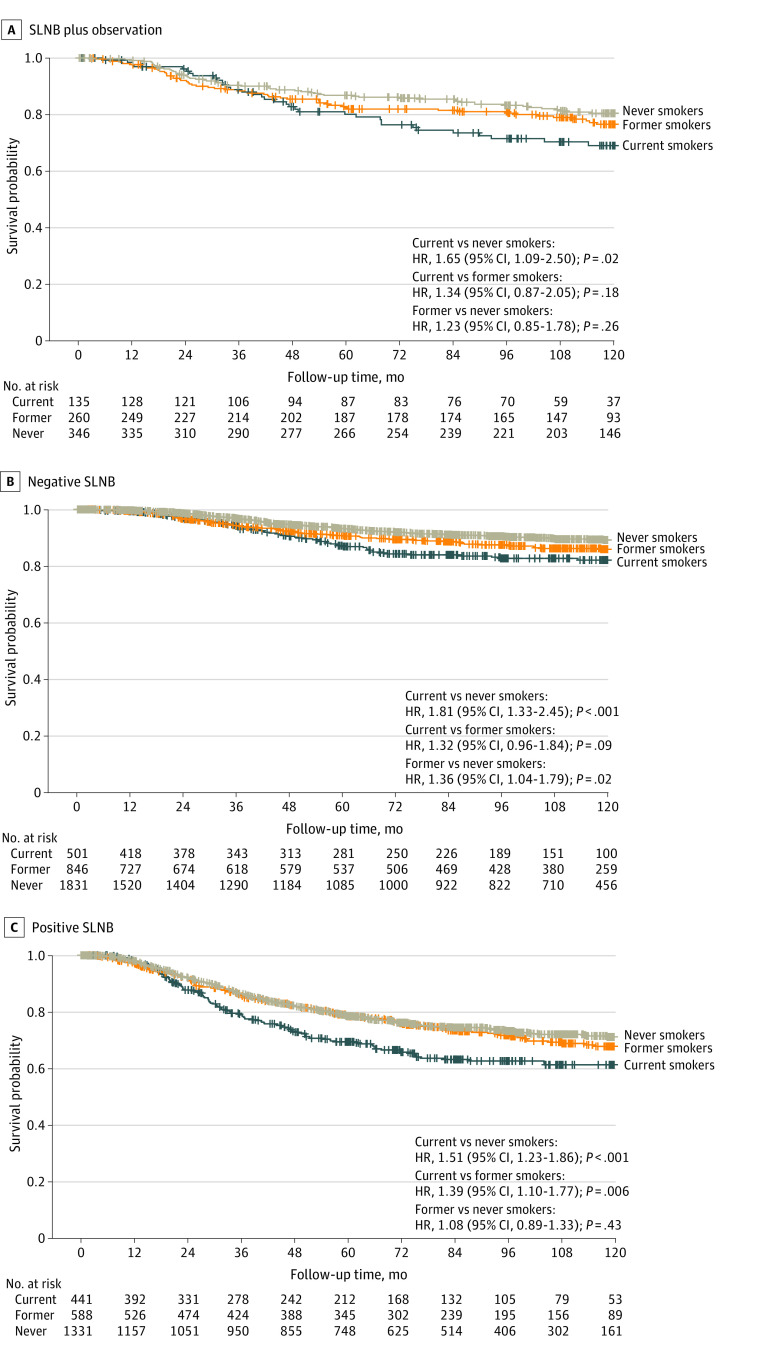
Melanoma-Specific Survival of Current, Former, and Never Smokers A, Patients who did not undergo sentinel lymph node biopsy (SLNB) (n = 741); overall *P* = .06. B, Patients with tumor-negative SLNB (n = 3178); overall *P* < .001. C, Patients with tumor-positive SLNB (n = 2360); overall *P* < .001. HR indicates hazard ratio.

Disease-specific survival of current, former, and never smokers was assessed within each nodal group as previously described. Figure 2A shows MSS for the SLN plus observation group. Among current smokers, there was a significantly increased risk of melanoma-associated death (MAD) compared with never smokers (hazard ratio [HR], 1.65 [95% CI, 1.09-2.50]; *P* = .02). There was no significant difference in MSS between current and former smokers (HR, 1.34 [95% CI, 0.87-2.05]; *P* = .18) or between former and never smokers (HR, 1.23 [95% CI, 0.85-1.78]; *P* = .26). Figure 2B demonstrates MSS for patients in the SLNB-negative group. Again, current smokers experienced a greater risk of MAD compared with never smokers (HR, 1.81 [95% CI, 1.33-2.45]; *P* < .001). The risk of MAD was also significant for former vs never smokers (HR, 1.36 [95% CI, 1.04-1.79]; *P* = .02) but not for current vs former smokers (HR, 1.32 [95% CI, 0.96-1.84]; *P* = .09). Figure 2C displays MSS for the SLNB-positive group. Current smokers experienced a greater risk of MAD compared with both former smokers (HR, 1.39 [95% CI, 1.10-1.77]; *P* = .006) and never smokers (HR, 1.51 [95% CI, 1.23-1.86]; *P* < .001), while the mortality risk for former and never smokers was similar (HR, 1.08 [95% CI, 0.89-1.33]; *P* = .43).

In a multivariable analysis of the overall study population, current smoking was associated with decreased MSS (HR, 1.48 [95% CI, 1.26- 1.75]; *P* < .001). By contrast, former smoking was not (HR, 1.03 [95% CI, 0.89-1.20]; *P* = .68) (eTable 1 in Supplement 1). When analyzed by nodal groups on multivariable analysis, current smoking was an independent risk factor for melanoma mortality across all 3 nodal groups, whereas former smoking was not (Table 2). There was no interaction term between smoking and age or smoking and sex.

**Table 2.** Multivariate Melanoma-Specific Survival by SLNB Status

Parameter	No SLNB (n = 741)		SLNB negative (n = 3178)		SLNB positive (n = 2360)		
	HR (95% CI)	*P* value	HR (95% CI)	*P* value	HR (95% CI)	*P* value
Age, y	1.02 (1.00-1.03)	.006	1.02 (1.01-1.03)	.002	1.01 (1.00-1.02)	.005
Sex						
Women	1 [Reference]	NA	1 [Reference]	NA	1 [Reference]	NA
Men	1.34 (0.92-1.96)	.13	1.21 (0.92-1.58)	.17	1.28 (1.06-1.54)	.01
Breslow thickness, mm	1.09 (1.06-1.13)	<.001	1.09 (1.06-1.12)	<.001	1.12 (1.09-1.14)	<.001
Ulceration						
Absent	1 [Reference]	NA	1 [Reference]	NA	1 [Reference]	NA
Present	1.75 (1.26-2.44)	.001	2.66 (2.09-3.39)	<.001	2.25 (1.89-2.67)	<.001
Primary site						
Extremity	1 [Reference]	NA	1 [Reference]	NA	1 [Reference]	NA
Head and/or neck	1.14 (0.68-1.90)	.61	1.53 (1.07-2.17)	.02	1.14 (0.87-1.50)	.34
Trunk	1.94 (1.31-2.88)	.001	1.57 (1.19-2.06)	.001	1.17 (0.97-1.42)	.09
Smoking status						
Current	1.68 (1.09-2.61)	.02	1.85 (1.35-2.52)	<.001	1.29 (1.04-1.59)	.02
Former	1.03 (0.70-1.51)	.89	1.20 (0.91-1.58)	.20	0.95 (0.78-1.17)	.65
Never	1 [Reference]	NA	1 [Reference]	NA	1 [Reference]	NA

Abbreviations: HR, hazard ratio; NA, not applicable; SLNB, sentinel lymph node biopsy.

The relative increased risk of melanoma-specific mortality in current smokers was greatest for patients in the SLNB-negative group (HR, 1.85 [95% CI, 1.35-2.52]; *P* < .001). This finding was consistent for MSLT-I (HR, 1.86 [95% CI, 1.18-2.94]; *P* = .008) and MSLT-II (HR, 1.75 [95% CI, 1.13-2.70]; *P* = .01) when the 2 trials were assessed independently (eTables 2 and 3 in Supplement 1). For patients in the SLNB-positive group, current smoking was an independent prognostic factor in the combined studies (HR, 1.29 [95% CI, 1.04-1.59]; *P* = .02) (Table 2) and in MSLT-II (eTables 2 and 3 in Supplement 1). The number of patients in the SLNB-positive group in MSLT-I was relatively small (n = 203), and there was no association between smoking status and survival. When patients in the SLNB-positive group were separated into those with RT-PCR and histopathologic assessments, there was no difference in smoking-associated mortality in the group undergoing RT-PCR (HR, 1.28 [95% CI, 0.38-2.07]; *P* = .69). In the histopathologic diagnosis group, the risk was significant (HR, 1.27 [95% CI, 1.03-1.58]; *P* = .03) (eTable 4 in Supplement 1). There was no interaction between smoking and age or sex, indicating that the association of smoking with diminished survival was neither age nor sex dependent.

### Quantitative Smoking and Survival

The quantitative association of smoking on the risk of MAD was evaluated. For 2586 of the 2771 current and former smokers with quantitative smoking data (93.3%) assessed together, there was no detectable association between the risk of MAD and the number of cigarettes smoked per day or years smoked on multivariable analysis (eTable 5 in Supplement 1); this was true regardless of whether smoking status was included as a variable in the analysis. When assessed by nodal groups, there was also no association between MAD and the number of cigarettes smoked per day or years smoked in either the SLN plus observation or the SLNB-negative groups. In the SLNB-positive group, former and current smokers who smoked 20 or more cigarettes/d had a greater risk of MAD (HR, 1.47 [95% CI, 1.05-2.06]; *P* = .03) (eTable 6 in Supplement 1).

**Figure 3.**
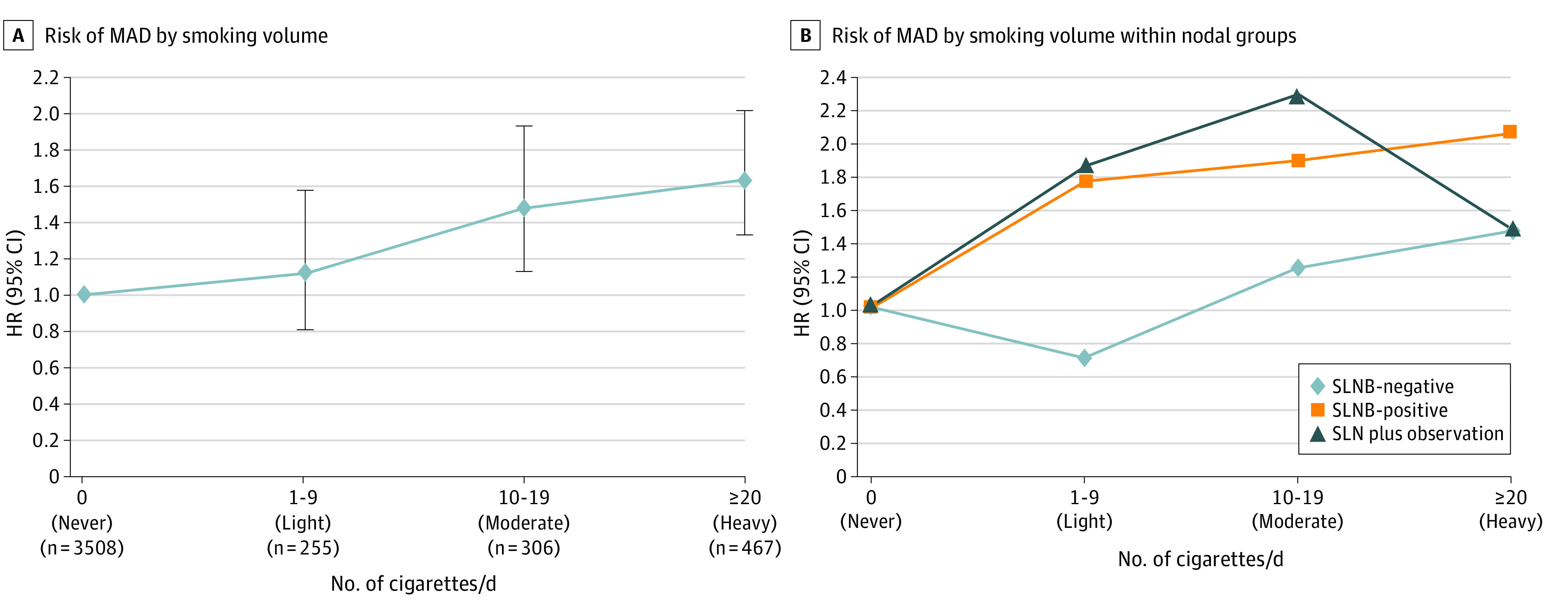
Adjusted Risk of Melanoma-Associated Death (MAD) in Current vs Never Smokers A, Data are stratified by smoking volume; B, by smoking volume within nodal groups (tumor-negative sentinel lymph node biopsy [SLNB-negative], SLNB-positive, and SNL plus observation). HR indicates hazard ratio.

Of the 1077 current smokers, 1028 (95.5%) had the number of cigarettes per day recorded. When current smokers were assessed by light (1-9 cigarettes/d), moderate (10-19 cigarettes/d) or heavy (≥20 cigarettes/d) smoking, for all 3 SLN groups combined, adjusted risk of MAD rose by approximately 20% for each of 3 quantitative smoking categories compared with nonsmokers (Figure 3A). Relative to nonsmokers, heavy smokers (HR, 1.63 [95% CI, 1.33-2.01]; *P* < .001) and moderate smokers (HR, 1.48 [95% CI, 1.13-1.93]; *P* = .004) had an increased risk of MAD, whereas light smokers did not (HR, 1.13 [95% CI, 0.81-1.58]; *P* = .47) (eTable 7 in Supplement 1). The difference between light and heavy smokers was significant (HR, 1.45 [95% CI, 1.00-2.09]; *P* = .05).

When assessed by each SLN group separately, patients in the SLNB-negative group who were heavy smokers had more than double the risk of MAD compared with nonsmoking patients (HR, 2.06 [95% CI, 1.36-3.13]; *P* < .001) (Figure 3B and eTable 8 in Supplement 1). This was also the case for moderate smokers who did not undergo SLNB (HR, 2.30 [95% CI, 1.10-4.80]; *P* = .03), although the patient number was limited in the moderate smoking SLN plus observation group (n = 35) (Figure 3B and eTable 8 in Supplement 1). In the SLNB-positive group, only heavy smokers had a significantly increased risk of melanoma mortality compared with nonsmokers (HR, 1.47 [95% CI, 1.12-1.92]; *P* = .005) (Figure 3B and eTable 8 in Supplement 1).

## Discussion

Using the prospectively maintained databases from 2 large randomized, multicenter and multinational trials, MSLT-I and MSLT-II, we found in this cohort study that smoking at the time of melanoma diagnosis was associated with shorter MSS across all SLN groups. For the SLNB-negative group, smoking was the second-highest risk factor for melanoma-specific death, second only to primary tumor ulceration, with an HR of 1.85 for current smokers relative to never smokers. Among patients in the SLNB-negative group who currently smoked 20 or more cigarettes per day, the risk of dying from their disease was more than double that of nonsmoking patients. Smoking was also associated with significantly decreased MSS in patients who underwent nodal observation, most of whom (approximately 80%) are presumed to be in the SLNB-negative group,^24^ and in patients in the positive-SLNB group. Smoking remained among the top 3 factors associated with increased melanoma-specific mortality in these 2 groups.

To our knowledge, this is the first report of increased risk of MAD in patients diagnosed with cutaneous melanoma who smoke on multivariable analysis inclusive of ulceration and stage.^12,18,19,25^ Reasons for mixed data among prior studies may include the limitations of retrospective reviews of large databases, as smoking is often underassessed at time of cancer diagnosis.^26,27,28^ For example, in the study by Mattila et al,^19^ only 68% of patients had smoking status available for analysis, whereas our database included smoking status for 90.2% of patients. Additionally, our study includes close follow-up of patients, expert histopathologic examination, well-documented surgical management, and frequent monitoring for MSS per the clinical trial protocols.

Previous work from the MSLT databases^16^ demonstrated that patients who smoke have a 27% likelihood of SLNB positivity, a significant increase from the 18% and 19% for never and former smokers, respectively. Congruent with other published reports, smokers were more likely to be in the positive-SLNB group and to have deeper and ulcerated primary tumors in our study. We demonstrate that patients in the SLNB-negative group had the greatest relative smoking-associated risk of MAD among the 3 SLNB groups. Thus, smoking may promote early regional and systemic dissemination from primary tumors.

There are multiple potential mechanisms for promotion of tumor metastasis and worse survival in patients with melanoma who smoke. Smoking has been proven to decrease cutaneous blood flow,^29,30^ cause endothelial injury, and induce a procoagulant state.^31,32^ In clinical studies of patients undergoing plastic surgery, skin flap necrosis is much more common in current but not former smokers,^33^ and smoking cessation even 1 week prior to surgery could mitigate flap loss.^34^ These studies indicate that skin is reversibly sensitive to the effects of smoking. Other studies^35,36,37,38,39^ have demonstrated multiple procancer effects of nicotine on tumor cells. Smoking derivative products in individuals is known to alter various immune responses, which may reduce host immune responses in controlling melanoma disease progression.^40^ Smoking has also been shown to increase testosterone levels, which appear to promote melanoma metastasis and contribute to immunotherapy resistance.^41,42,43^

The association of persistent smoking vs smoking cessation was not specifically addressed in this study. However, the absence of an overall negative survival association with former smoking in multivariable analysis supports the hypothesis that any negative effect from smoking could be largely reversible. The known reversible effects of smoking on cortisol levels, DNA damage, and risk of clinical complications could all be implicated as contributors to poor outcomes in patients with melanoma and thus mitigated by smoking cessation.^42,44,45^ While further studies evaluating the effect of smoking cessation on melanoma mortality risk are needed, patients with early-stage melanoma should be strongly encouraged to quit as a potential mitigation strategy for disease progression.

In our cohort, few patients underwent treatment with modern systemic therapies,^24^ which allows a more direct assessment of disease biology but may underestimate the implications of smoking effects on patients in the era of immunotherapy. Multiple studies^46,47,48^ have confirmed that in patients with lung cancer undergoing systemic immune checkpoint inhibitor (ICI) therapy, those who smoke have an increased survival and better response rate to ICI compared with nonsmokers. Proposed mechanisms include increased programmed cell death ligand 1 expression and increased tumor mutational burden in non–small cell lung tumors of smokers.^49,50,51^ In patients with melanoma, the reports of smoking and response to ICI are limited. One study by Zhang et al^52^ found that a previously described smoking-related gene signature identified in patients with melanoma receiving ICI portended significantly worse overall response, disease control rates, and progression-free and overall survival. This suggests any adverse effects of smoking on outcomes for patients with melanoma would be amplified in the current therapeutic era.

This prospective study analysis cannot prove a causal relationship between smoking tobacco products and MAD. It is possible that smoking-associated behaviors such as alcohol consumption and marijuana use, which were not captured in the MSLT databases, contributed to the increased risk observed. However, in the analysis by Hardie et al,^18^ smoking was independently associated with shorter MSS on multivariate analysis when examined with factors such as alcohol consumption, socioeconomic status, and vitamin D levels. Additionally, the appearance of a dose-response trend for current smokers in our study, the plausibility of the suggested mechanisms for smoking’s effect and reversal of the negative survival association in former smokers implicate smoking as a cause for diminished survival of melanoma. As our analysis focused on MSS, any competing mortality due to other smoking-related illnesses would tend to diminish the association we observed. The presence of the strong observed association, despite this potential confounder, argues for the significance of smoking specifically in relationship to melanoma progression.

Future clinical trials should consider including smoking status as a stratification factor, and further work characterizing gene expression profiling as it relates to clinical smoking should be pursued. Larger studies are needed to confirm the dose-response pattern suggested for current smokers in this study and to provide further follow-up and adjuvant treatment guidance. Also, future studies are needed to assess the benefit of smoking cessation following a melanoma diagnosis, which was not captured in either MSLT study.

### Limitations

Our study has some limitations. By combining 2 different trials, we created a group of patients (SLNB-positive) with heterogenous subsequent treatments (ie, completion dissection vs observation after SLNB positivity in the MSLT-II cohort). However, the strongest association with smoking and decreased MSS was in patients in the SLNB-negative group, all of whom would have undergone subsequent nodal surveillance without additional surgery. Furthermore, the association between smoking and MSS in the SLNB-negative group held true when trials were analyzed separately.

Other limitations include a single time point and self-reported smoking status, although these methods have been used historically, and patients with nonpulmonary disease have demonstrated high fidelity with biochemical confirmatory testing.^53,54^ Serial assessments of smoking and use of biochemical testing would have strengthened this report. Additionally, the former smoking group was defined as quitting smoking any time prior to trial enrollment, which likely creates a heterogenous group of individuals that varied from recent to remote smoking cessation and limits conclusions about that cohort. Finally, as noted above, patients in these trials did not have access to current checkpoint blockade or targeted adjuvant therapies, whose use might affect these observations.

## Conclusions

The findings of this cohort study of patients with clinical stage I and II primary cutaneous melanoma suggest that smoking at the time of diagnosis was associated with increased risk of MAD. Because smoking could be considered a risk factor for disease progression, increased vigilance in the management of patients who smoke may be warranted. Quantitative smoking data should be included in melanoma databases, and inclusion of smoking as a stratification factor in clinical trials should be considered. Although the association of continued smoking was not specifically addressed in this study, it seems prudent to recommend smoking cessation to patients with melanoma at the time of diagnosis.

## Notes

Supplement 1. **eTable 1.** Multivariate MSS, Overall Study Population (N = 6279) **eTable 2.** Multivariate MSS by SLNB Status, MSLT1 **eTable 3.** Multivariate MSS by SLNB Status, MSLT2 **eTable 4.** Multivariate MSS by RT-PCR vs Histopathologic Analysis **eTable 5.** Multivariate MSS in Current and Former Smokers **eTable 6.** Multivariate MSS by SLNB Status in Current and Former Smokers **eTable 7.** Multivariate MSS in Current and Never Smokers **eTable 8.** Multivariate MSS by SLNB Status in Current and Never Smokers

Supplement 2.
Data Sharing Statement
