# Supplementary material for: Smoking Status and Survival in Patients With Early-Stage Primary Cutaneous Melanoma
Source: JAMA Netw Open. 2024 Feb 6;7(2):e2354751. doi: 10.1001/jamanetworkopen.2023.54751 (PMC10848058; doi:10.1001/jamanetworkopen.2023.54751)
Supplement: Supplement 1. — eTable 1. Multivariate MSS, Overall Study Population (N = 6279) eTable 2. Multivariate MSS by SLNB Status, MSLT1 eTable 3. Multivariate MSS by SLNB Status, MSLT2 eTable 4. Multivariate MSS by RT-PCR vs Histopathologic Analysis eTable 5. Multivariate MSS in Current and Former Smokers eTable 6. Multivariate MSS by SLNB Status in Current and Former Smokers eTable 7. Multivariate MSS in Current and Never Smokers eTable 8. Multivariate MSS by SLNB Status in Current and Never Smokers [file jamanetwopen-e2354751-s001.pdf]

Supplementary Online Content

Jackson KM, Jones PC, Fluke LM, et al. Smoking status and survival in patients with early-stage primary cutaneous melanoma. *JAMA Netw Open*. 2024;7(2):e2354751. doi:10.1001/jamanetworkopen.2023.54751

eTable 1. Multivariate MSS, Overall Study Population (N = 6279)

eTable 2. Multivariate MSS by SLNB Status, MSLT1

eTable 3. Multivariate MSS by SLNB Status, MSLT2

eTable 4. Multivariate MSS by RT-PCR vs Histopathologic Analysis

eTable 5. Multivariate MSS in Current and Former Smokers

eTable 6. Multivariate MSS by SLNB Status in Current and Former Smokers

eTable 7. Multivariate MSS in Current and Never Smokers

eTable 8. Multivariate MSS by SLNB Status in Current and Never Smokers

This supplementary material has been provided by the authors to give readers additional information about their work.

© 2024 Jackson KM et al. *JAMA Network Open*.

eTable 1. Multivariate MSS, Overall Study Population (N = 6279)

Parameter	All Patients (n=6279)		
	**HR**	**95% CI**	**p-value**
**Age, years**	1.01	(1.01-1.02)	<0.001
**Gender**			
Female (ref)	1.00		
Male	1.29	(1.12-1.48)	<0.001
**Breslow, mm**	1.10	(1.08-1.12)	<0.001
**Ulceration**			
Absent (ref)	1.00		
Present	2.27	(1.99-2.58)	<0.001
**Primary Site**			
Extremity (ref)	1.00		
Head/Neck	1.23	(1.01-1.49)	0.042
Trunk	1.37	(1.18-1.58)	<0.001
**Smoking Status**			
Current	1.48	(1.26-1.75)	<0.001
Former	1.03	(0.89-1.20)	0.678
Never (ref)	1.00		
**SLNB Status**			
No SLNB	1.73	(1.41-2.11)	<0.001
SLNB Negative (ref)	1.00		
SLNB Positive	2.46	(2.13-2.85)	<0.001

eTable 2. Multivariate MSS by SLNB Status, MSLT1

Parameter	No SLNB (n=741)			SLNB Negative (n=876)			SLNB Positive (n=203)		
	**HR**	**95% CI**	**p-value**	**HR**	**95% CI**	**p-value**	**HR**	**95% CI**	**p-value**
**Age, years**	1.02	(1.00-1.03)	0.006	1.01	(1.00-1.03)	0.077	1.02	(1.00-1.04)	0.053
**Gender**									
Female (ref)	1.00			1.00			1.00		
Male	1.34	(0.92-1.96)	0.131	1.00	(0.68-1.46)	0.996	1.23	(0.76-2.01)	0.404
**Breslow, mm**	1.09	(1.06-1.13)	<0.001	1.19	(1.10-1.28)	<0.001	1.06	(0.96-1.17)	0.290
**Ulceration**									
Absent (ref)	1.00			1.00			1.00		
Present	1.75	(1.26-2.44)	0.001	2.04	(1.44-2.90)	<0.001	2.14	(1.34-3.41)	0.002
**Primary Site**									
Extremity (ref)	1.00			1.00			1.00		
Head/Neck	1.14	(0.68-1.90)	0.612	1.33	(0.80-2.22)	0.274	1.73	(0.84-3.54)	0.134
Trunk	1.94	(1.31-2.88)	0.001	1.90	(1.28-2.82)	0.001	1.19	(0.71-1.98)	0.510
**Smoking Status**									
Current	1.68	(1.09-2.61)	0.019	1.86	(1.18-2.94)	0.008	1.14	(0.65-2.01)	0.652
Former	1.03	(0.70-1.51)	0.888	1.09	(0.73-1.62)	0.681	0.76	(0.45-1.28)	0.299
Never (ref)	1.00			1.00			1.00		

© 2024 Jackson KM et al. *JAMA Network Open*.

eTable 3. Multivariate MSS by SLNB Status, MSLT2

Parameter	SLNB Negative (n=2302)			SLNB Positive (n=2157)		
	HR	95% CI	p-value	HR	95% CI	p-value
**Age, years**	1.02	(1.00-1.03)	0.008	1.01	(1.00-1.02)	0.012
**Gender**						
Female (ref)	1.00			1.00		
Male	1.40	(0.95-2.06)	0.085	1.28	(1.04-1.57)	0.018
**Breslow, mm**	1.08	(1.04-1.12)	<0.001	1.12	(1.09-1.15)	<0.001
**Ulceration**						
Absent (ref)	1.00			1.00		
Present	3.05	(2.17-4.28)	<0.001	2.29	(1.90-2.76)	<0.001
**Primary Site**						
Extremity (ref)	1.00			1.00		
Head/Neck	1.71	(1.05-2.79)	0.031	1.10	(0.82-1.48)	0.529
Trunk	1.48	(1.00-2.18)	0.049	1.19	(0.97-1.45)	0.099
**Smoking Status**						
Current	1.75	(1.13-2.70)	0.012	1.28	(1.02-1.61)	0.033
Former	1.17	(0.79-1.72)	0.436	0.95	(0.76-1.19)	0.664
Never (ref)	1.00			1.00		

© 2024 Jackson KM et al. *JAMA Network Open*.

eTable 4. Multivariate MSS by RT-PCR vs Histopathologic Analysis

Parameter	RT-PCR Pos (n=221)			Histopathologic Pos (n=2139)		
	**HR**	**95% CI**	**p-value**	**HR**	**95% CI**	**p-value**
**Age, years**	1.01	(0.98-1.05)	0.549	1.01	(1.00-1.02)	0.003
**Gender**						
Female (ref)	1.00			1.00		
Male	1.89	(0.65-5.51)	0.244	1.24	(1.03-1.50)	0.026
**Breslow, mm**	1.02	(0.78-1.33)	0.890	1.11	(1.09-1.14)	<0.001
**Ulceration**						
Absent (ref)	1.00			1.00		
Present	2.37	(1.01-5.56)	0.048	2.17	(1.81-2.59)	<0.001
**Primary Site**						
Extremity (ref)	1.00			1.00		
Head/Neck	2.59	(0.69-9.71)	0.158	1.28	(0.96-1.72)	0.093
Trunk	2.68	(0.68-10.6)	0.160	1.16	(0.96-1.40)	0.128
**Smoking Status**						
Current	1.28	(0.38-2.07)	0.690	1.27	(1.03-1.58)	0.028
Former	0.59	(0.21-1.68)	0.320	0.97	(0.79-1.20)	0.800
Never (ref)	1.00			1.00		

© 2024 Jackson KM et al. *JAMA Network Open*.

eTable 5. Multivariate MSS in Current and Former Smokers

Parameter	Current/Former Smokers (n=2586)		
	**HR**	**95% CI**	**p-value**
**Age, years**	1.02	(1.01-1.03)	<0.001
**Gender**			
Female (ref)	1.00		
Male	1.12	(0.91-1.39)	0.290
**Breslow, mm**	1.13	(1.10-1.16)	<0.001
**Ulceration**			
Absent (ref)	1.00		
Present	2.17	(1.80-2.62)	<0.001
**Primary Site**			
Extremity (ref)	1.00		
Head/Neck	1.26	(0.95-1.67)	0.110
Trunk	1.41	(1.14-1.74)	0.002
**SLNB Status**			
No SLNB	1.57	(1.20-2.06)	0.001
SLNB Negative (ref)	1.00		
SLNB Positive	2.05	(1.66-2.54)	<0.001
**Smoking Status**			
Current	1.48	(1.18-1.86)	<0.001
Former (ref)	1.00		
**# Cigarettes/day**			
1-9 (ref)	1.00		
10-19	1.12	(0.86-1.46)	0.387
20+	1.20	(0.94-1.52)	0.146
**# Years Smoked**	1.00	(0.99-1.00)	0.334

© 2024 Jackson KM et al. *JAMA Network Open*.

eTable 6. Multivariate MSS by SLNB Status in Current and Former Smokers

Parameter	No SLNB (n=386)			SLNB Negative (n=1246)			SLNB Positive (n=954)		
	HR	95% CI	p-value	HR	95% CI	p-value	HR	95% CI	p-value
**Age, years**	1.00	(0.98-1.02)	0.878	1.02	(1.00-1.03)	0.056	1.02	(1.01-1.03)	0.002
**Gender**									
Female (ref)	1.00			1.00			1.00		
Male	1.24	(0.73-2.10)	0.423	1.08	(0.72-1.60)	0.713	1.09	(0.82-1.46)	0.552
**Breslow, mm**	1.22	(1.13-1.33)	<0.001	1.16	(1.09-1.24)	<0.001	1.11	(1.08-1.15)	<0.001
**Ulceration**									
Absent (ref)	1.00			1.00			1.00		
Present	1.72	(1.12-2.66)	0.014	2.79	(1.98-3.94)	<0.001	1.98	(1.53-2.58)	<0.001
**Primary Site**									
Extremity (ref)	1.00			1.00			1.00		
Head/Neck	1.74	(0.90-3.34)	0.099	1.34	(0.79-2.26)	0.278	1.03	(0.68-1.55)	0.883
Trunk	2.36	(1.34-4.16)	0.003	1.75	(1.19-2.57)	0.004	1.13	(0.85-1.50)	0.401
**Smoking Status**									
Current	1.29	(0.75-2.24)	0.360	1.64	(1.08-2.49)	0.020	1.49	(1.10-2.03)	0.011
Former (ref)	1.00			1.00					
**# Cigarettes/day**									
1-9 (ref)	1.00			1.00			1.00		
10-19	0.59	(0.32-1.10)	0.096	1.32	(0.81-2.14)	0.266	1.19	(0.83-1.73)	0.345
20+	0.63	(0.37-1.06)	0.085	1.18	(0.74-1.88)	0.492	1.47	(1.05-2.06)	0.025
**# Years Smoked**	1.01	(0.99-1.03)	0.206	1.00	(0.98-1.02)	0.904	0.99	(0.98-1.00)	0.033

© 2024 Jackson KM et al. *JAMA Network Open*.

eTable 7. Multivariate MSS in Current and Never Smokers

Parameter	Current/Never Smokers (n=4536)		
	**HR**	**95% CI**	**p-value**
**Age, years**	1.02	(1.01-1.02)	<0.001
**Gender**			
Female (ref)	1.00		
Male	1.29	(1.09-1.52)	0.003
**Breslow, mm**	1.09	(1.07-1.11)	<0.001
**Ulceration**			
Absent (ref)	1.00		
Present	2.34	(2.00-2.73)	<0.001
**Primary Site**			
Extremity (ref)	1.00		
Head/Neck	1.18	(0.93-1.50)	0.167
Trunk	1.31	(1.10-1.56)	0.002
**SLNB Status**			
No SLNB	1.76	(1.37-2.26)	<0.001
SLNB Negative (ref)	1.00		
SLNB Positive	2.52	(2.11-2.99)	<0.001
**# Cigarettes/day**			
0 (ref)			
1-9	1.13	(0.81-1.58)	0.471
10-19	1.48	(1.13-1.93)	0.004
20+	1.63	(1.33-2.01)	<0.001

© 2024 Jackson KM et al. *JAMA Network Open*.

eTable 8. Multivariate MSS by SLNB Status in Current and Never Smokers

Parameter	No SNB (n=478)			SNB Negative (n=2304)			SNB Positive (n=1754)		
	HR	95% CI	p-value	HR	95% CI	p-value	HR	95% CI	p-value
**Age, years**	1.03	(1.01-1.04)	0.002	1.02	(1.00-1.02)	0.039	1.01	(1.00-1.02)	0.021
**Gender**									
Female (ref)	1.00			1.00			1.00		
Male	1.51	(0.95-2.40)	0.084	1.11	(0.81-1.51)	0.515	1.31	(1.05-1.62)	0.015
**Breslow, mm**	1.09	(1.05-1.13)	<0.001	1.08	(1.04-1.12)	<0.001	1.10	(1.07-1.14)	<0.001
**Ulceration**									
Absent (ref)	1.00			1.00			1.00		
Present	1.84	(1.22-2.79)	0.004	2.36	(1.76-3.17)	<0.001	2.46	(2.00-3.02)	<0.001
**Primary Site**									
Extremity (ref)	1.00			1.00			1.00		
Head/Neck	0.93	(0.48-1.81)	0.828	1.62	(1.06-2.48)	0.026	1.09	(0.79-1.50)	0.614
Trunk	1.78	(1.10-2.88)	0.018	1.44	(1.04-2.01)	0.030	1.19	(0.96-1.48)	0.120
**# Cigarettes/day**									
0 (ref)	1.00			1.00			1.00		
1-9	1.86	(0.88-3.94)	0.106	1.76	(1.04-2.98)	0.035	0.70	(0.41-1.19)	0.189
10-19	2.30	(1.10-4.80)	0.026	1.89	(1.12-3.19)	0.017	1.24	(0.88-1.74)	0.225
20+	1.49	(0.84-2.63)	0.173	2.06	(1.36-3.13)	<0.001	1.47	(1.12-1.92)	0.005

© 2024 Jackson KM et al. *JAMA Network Open*.
