# Supplementary material for: Smoking Status and Survival in Patients With Early-Stage Primary Cutaneous Melanoma
Source: JAMA Netw Open. 2024 Feb 6;7(2):e2354751. doi: 10.1001/jamanetworkopen.2023.54751 (PMC10848058; doi:10.1001/jamanetworkopen.2023.54751)
Supplement: Supplement 2. — Data Sharing Statement [file jamanetwopen-e2354751-s002.pdf]

Data Sharing Statement

Jackson. Smoking Status and Survival in Patients With Early-Stage Primary Cutaneous Melanoma. *JAMA Netw Open*. Published February 06, 2024.
doi:10.1001/jamanetworkopen.2023.54751

Data
Data available: No
